# Comprehensive analysis reveals XCL2 as a cancer prognosis and immune infiltration-related biomarker

**DOI:** 10.18632/aging.205156

**Published:** 2023-10-30

**Authors:** Wu Chen, Fan Zou, Tianbao Song, Yuqi Xia, Ji Xing, Ting Rao, Xiangjun Zhou, Jinzhuo Ning, Sheng Zhao, Weimin Yu, Fan Cheng

**Affiliations:** 1 Department of Urology, Renmin Hospital of Wuhan University, Wuhan 430060, China

**Keywords:** X-C Motif Chemokine Ligand 2, pan-cancer analysis, bioinformatics, immune infiltration, immunotherapy

## Abstract

Background: X-C Motif Chemokine Ligand 2 (XCL2) is a 114 amino acid, structurally conserved chemokine involved in activating cytotoxic T cells. However, the pathophysiological mechanisms of XCL2 protein in various disease conditions, particularly cancer, remain poorly understood.

Methods: Bioinformatics was used to detect the expression of XCL2, the relationship between survival time and XCL2 in BLCA patients, the mutational status of XCL2, the role of XCL2 in the tumor immune microenvironment, and the sensitivity of XCL2-targeted drugs in 33 cancers. *In vitro* experiments were conducted to investigate the chemotactic effects of XCL2 expression on M1-type macrophages in human specimens and in isolated cancer cells.

Results: XCL2 expression was downregulated in tumor tissues and closely associated with the prognosis of human cancers. Furthermore, XCL2 affects DNA methylation, tumor mutation burden (TMB), microsatellite instability (MSI), and mismatch repair (MMR) in human cancers. The expression level of XCL2 significantly correlated with infiltrated immune cells, immunological pathways, and other immune markers. More importantly, we found that XCL2 was positively associated with T lymphocytes and macrophages in the transcriptome and single-cell sequencing data. Using multiple immunofluorescence staining, we found that the expression level of XCL2 was upregulated in many cells in pan-cancer samples, and the number of M1 macrophage marker CD68 and INOS-positive cells increased. 786O, U251, and MDA-MB-231 cells could recruit more M1 macrophages *in vitro* after overexpressing XCL2.

Conclusions: Our results reveal that XCL2 could act as a vital chemokine in pan-cancer and provide new targets and concepts for cancer treatment.

## INTRODUCTION

According to the World Health Organization (WHO) report from 2019 [1], cancer has become the primary or second leading cause of disease in 112 of 183 countries with the highest number of deaths before age 70, ranking third or fourth in the other 23 countries. It also showed a new peak in incidence and mortality compared to 2018 [2]. Over the past few years, the advent of immunotherapy has offered new ideas for treating cancer progression and metastasis. Therefore, the importance of the tumor microenvironment is currently the most studied and difficult aspect of cancer research [3]. The identification of novel immune checkpoints such as PD-1, PD-L1, CTLA-4, TIM3, and others by inciting or suppressing cytotoxic CD8+ T lymphocytes, regulatory T cells, NK cells, and others has provided new ideas for the treatment of malignant tumors [4–6]. Single-agent immune checkpoint inhibitor (ICI) therapy produces long-term responses in patients with advanced cancer [7]. However, only a small proportion of patients benefit, and recurrence is common owing to multiple resistance mechanisms [8]. The molecular mechanism of cancer cell death by activating numerous immune cells remains unclear. Identifying novel immunotherapeutic targets and biomarkers for cancer is vital for increasing cancer survival rates.

The chemokine family comprises a group of basic, structurally related molecules that regulate the cellular transport of different types of leukocytes [9]. Chemokines are classified into four subtypes based on the arrangement of the N-terminal two cysteine residues: CXC, CC, XC, and CX3C [10]. X-C Motif Chemokine Ligand 1 (XCL1), a C-class chemokine also known as lymphotactin, is a Protein Coding gene that is involved in several processes, including cytotoxic immune response [11], regulation of T lymphocyte development [12], and positive regulation of T cell chemotaxis [10]. Related studies [12] revealed that XCL1 mediates myeloid accumulation of thymic dendritic cells in the thymus, regulates T-cell development, and plays a role in establishing self-tolerance. In contrast to the well-studied XCL1, its paralog X-C Motif Chemokine Ligand 2 (XCL2) is a structurally conserved chemokine [13, 14] of 114 amino acids that has received less attention. Preliminary research indicates that XCL2, like XCL1, is involved in the development and activation of cytotoxic T cells [15] and the progression of malignant tumor growth [16]. Similarly, the pathophysiological mechanisms of the XCL2 protein in several disease conditions, particularly cancer, remain poorly understood.

Chemokine-related receptor and ligand genes remain popular worldwide, and the CXCL12/CXCR4/CXCR7 signaling pathway is the most widely studied, playing an important role in tumor progression, angiogenesis, cell metastasis, and survival, the effects of XCL2, an important member of the chemokine family, on human cancer have rarely been systematically explored and studied. XCL2 plays a significant role in the development and progression of various cancers. Based on previous studies of XCL1 and XCL2, we hypothesized that XCL2 might be involved in the formation and accumulation of immune cells such as T lymphocytes, B lymphocytes, and macrophages during the progression of tumor cells. Therefore, in the present study, we performed a comprehensive pan-cancer analysis based on several well-known open databases and loss-of-function assays to describe the profile of XCL2, including survival prognosis analysis, genetic alterations, methylation, immune infiltration, and drug sensitivity. These results suggest that XCL2 can be used as a therapeutic and prognostic pan-cancer biomarker.

## MATERIALS AND METHODS

### Data acquisition and differential expression analysis of XCL2

Briefly, RNA sequencing data in the transcripts per million reads format (TPM) of 33 types of cancer from TCGA and GTEx datasets were obtained from UCSC Xena database (https://xenabrowser.net/datapages/). RNA sequencing data were normalized to log2 (TPM + 1). The UCSC Xena database obtained clinical information such as survival time and tumor-node-metastasis (TNM) stage. Statistical analyses were performed using R software (version 4.2.1). The “Wilcox test” algorithm was used to compare XCL2 expression between tumor samples and adjacent normal tissues in 33 types of cancer, and differences with p < 0.05 were considered statistically significant. We visualized the data and generated violin graphs using Sangerbox (http://vip.sangerbox.com/home.html) and Xiantao Academic (https://www.xiantao.love/products/apply). Box diagrams were plotted using the “ggpubr” R package.

The 33 cancer types included the following: Adrenocortical carcinoma (ACC), Bladder urothelial carcinoma (BLCA), Breast invasive carcinoma (BRCA), Cervical squamous cell carcinoma and endocervical adenocarcinoma (CESC), Cholangiocarcinoma (CHOL), Colon adenocarcinoma (COAD), Lymphoid neoplasm diffuse large B-cell lymphoma (DLBC), Esophageal carcinoma (ESCA), Glioblastoma multiforme (GBM), Head and neck squamous cell carcinoma (HNSC), Kidney chromophobe (KICH), Kidney renal clear cell carcinoma (KIRC), Kidney renal papillary cell carcinoma (KIRP), Acute myeloid leukemia (LAML), Brain lower grade glioma (LGG), Liver hepatocellular carcinoma (LIHC), Lung adenocarcinoma (LUAD), Lung squamous cell carcinoma (LUSC), Mesothelioma (MESO), Ovarian serous cystadenocarcinoma (OV), Pancreatic adenocarcinoma (PAAD), Pheochromocytoma and Paraganglioma (PCPG), Prostate adenocarcinoma (PRAD), Rectum adenocarcinoma (READ), Sarcoma (SARC), Skin cutaneous melanoma (SKCM), Stomach adenocarcinoma (STAD), Testicular germ cell tumors (TGCT), Thyroid carcinoma (THCA), Thymoma (THYM), Uterine corpus endometrial carcinoma (UCEC), Uterine carcinosarcoma (UCS), and Uveal melanoma (UVM).

### Clinical correlation analysis of XCL2

Using the median XCL2 expression level as the cut-off value, patients in TCGA database were divided into two groups: those with high XCL2 expression and those with low XCL2 expression. The impact of XCL2 expression on overall survival (OS), disease-specific survival (DSS), disease-free survival (DFS), and progression-free interval (PFI) in 33 types of cancers was then analyzed and visualized using forest plots and Kaplan-Meier curves with the ‘‘survival” and “survminer” R packages. The hazard ratio (HR) was over 1 (HR > 1), indicating that XCL2 served as a risk factor for patient survival.

### Genetic alteration analysis of XCL2

In this study, we searched the cBioPortal database [17] (https://www.cbioportal.org/) for XCL2 genetic alteration information using the “Cancer Types Summary,” “Mutations,” and “Plots” modules, including the mutation sites, mutation types, and mutation counts. Additionally, we used Gene Set Cancer Analysis [18] (GSCA) (http://bioinfo.life.hust.edu.cn/web/GSCALite/), an online gene set cancer analysis platform, to explore the association between XCL2 expression and gene copy number variation (CNV) and degree of DNA methylation. We also entered four genes closely related to XCL2 function on GSCA website: XCL1, CXCL1, CXCL2, CXCL3, and CXCL4. Finally, we used Spearman’s correlation analysis to evaluate the relationship between XCL2 expression and five methyltransferases (DNMT1, TRDMT1, DNMT3A, DNMT3B, and DNMT3L) and five MMR-related genes (MLH1, MSH2, MSH6, EPCAM, and PMS2) in 33 types of cancers based on gene expression in the TCGA database. Statistical significance was set at P < 0.05.

### XCL2 expression in different molecular and immune subtypes of cancers

TISIDB database [19] is an online analytical website that synthesizes numerous databases and investigates the interactions between multiple cancers and immunity from various perspectives. The “Subtype” module in TISIDB database was used to study the relationship between XCL2 expression and molecular or immune subtypes in 33 types of cancers. The immune subtypes were C1 (wound healing), C2 (IFN-gamma dominant), C3 (inflammatory), C4 (lymphocyte depleted), C5 (immunologically quiet), and C6 (TGF-b dominant). Statistical significance was set at P < 0.05.

### Immune-related characteristics analysis of XCL2

First, we used ESTIMATE algorithm from R package to calculate the correlation of XCL2 expression with immunological score, stromal score, ESTIMATE score, and tumor purity across 33 cancers [20]. We downloaded the infiltration scores of various immune cells, including B cells, CD4+ T cells, CD8+ T cells, macrophages, neutrophils, and dendritic cells from TIMER2.0 database (http://timer.cistrome.org/). Spearman’s correlation analysis was used to evaluate the correlation between XCL2 expression and the infiltration of different types of immune cells. Furthermore, TMB and microsatellite instability (MSI) scores of each cancer were analyzed using TCGA database, and the correlation between XCL2 expression and TMB or MSI was calculated using Spearman correlation analysis. The “ssGSEA” algorithm from the “GSVA” R package was used to explore the correlation between XCL2 expression and tumor-infiltrating lymphocytes, immunostimulators, immune inhibitors, MHC molecules, chemokines, and chemokine receptors in 33 types of cancers.

### Correlation analysis of XCL2 and drug response and immunotherapy response

The drug sensitivity data were downloaded from the CellMiner database (http://discover.nci.nih.gov/cellminer/). The data were processed and visualized using the “impute,” “limma,” “ggplot2,” and “ggpubr” R packages. We calculated the correlation between XCL2 expression and drug response sensitivity using Spearman’s method. Additionally, immunotherapy response was predicted using TIDE database (http://tide.dfci.harvard.edu) and ROC Plotter (http://www.rocplot.org/).

### Protein-protein interaction (PPI) network analyses of XCL2

The data on potential protein interactions with XCL2 were downloaded from STRING database (https://string-db.org/). All data were imported into Cytoscape (v3.8.2) for analysis and visualization. We identified key modules and presented the top 50 and 10 nodes ranked by the MCC of cytoHubba using cytoHubba plugins. In addition, we used Spearman’s correlation analysis to explore the correlation of the top 10 genes in pan-cancer.

### Functional enrichment analysis of XCL2

The top 10 genes screened by Cytoscape were XCL1, XCL2, CCR1, CCR2, CCR3, CCR5, CCR7, XCR1, CXCR3, and CXCR6. The results of the Gene Ontology (GO) function and Kyoto Encyclopedia of Genes and Genomes (KEGG) enrichment analyses of the top 10 genes were downloaded from DAVID database (https://david.ncifcrf.gov/summary.jsp). The results were visualized using the BioLadder (https://www.bioladder.cn/web/#/chart/28) online mapping platform. Additionally, we downloaded gene ontology sets (c5.go.v7.5.1. symbols.gmt) and curated gene sets (c2.cp.kegg.v7.5.1. symbols.gmt) from the Gene Set Enrichment Analysis website (GSEA) (https://www.gsea-msigdb.org/gsea/downloads.jsp). The GO and KEGG enrichment analysis of XCL2 were conducted using “limma,” “org.Hs.eg.db,” “DOSE,” “clusterProfiler,” and “enrichplot” R packages. The analysis results were visualized using Xiantao Academic (https://www.xiantao.love/products/apply). We also analyzed the relationship between XCL2 and well-known cancer-related pathways activated or inhibited in pan-cancer using GSCALite database (https://bioinfo.life.hust.edu.cn/web/GSCALite/). The above pathways included apoptosis, cell cycle, DNA Damage Response (DDR), epithelial–mesenchymal transition (EMT), Hormone AR, Hormone ER, PI3K/AKT, RAS/MAPK, RTK, and TSC/mTOR.

### Single-cell RNA sequencing analysis

We analyzed the correlation between XCL2 expression and various cancer cell types using TISCH2 website (http://tisch.comp-genomics.org/home/), an scRNA-seq database focusing on the tumor microenvironment (TME). Furthermore, we obtained hallmark and single-cell signature analysis results for different cell types through the “GSEA” section of the “Dataset” module.

### Cell culture

Human BRCA cell line MDA-MB-231 and GBM cell line U251 were cultured in Dulbecco’s modified eagle medium (DMEM) containing 10% fetal bovine serum (FBS; Sijiqing Biological Engineering Materials Company, Hangzhou, China) in a humidified atmosphere containing 5% CO_2_ at 37° C, and KIRC cell line 786O were cultured in the RPMI-1640 medium (HyClone, USA). The human monocyte cell line THP-1 was cultured in RPMI-1640 media (Invitrogen, USA) supplemented with 10% FBS at 37° C in 5% CO_2_. Firstly, THP1 cells were stimulated for six hours with 320 nM phorbol 12-myristate 13-acetate (PMA; Sigma, USA) to differentiate into M0 macrophages. M0 macrophages were then treated with 100 ng/mL Lipopolysaccharide (LPS; Beyotime, Shanghai, China) and incubated at 37° C for 48 h to induce M1 macrophage polarization.

### Lentivirus infection

Lentivirus was purchased from OBiO Technology (Shanghai, China). The lentiviral expression vector designated XCL2-OE (pSLenti-EF1-P2A-Puro-CMV-XCL2-3xFlag-WPRE) was used for XCL2 gene (NM_003175.4) delivery and stable overexpression. The empty vector designated as XCL2-NC (pSLenti-EF1-P2A-Puro-CMV-MCS-3xFlag-WPRE) was used as a negative control. The MOI (multiplicity of the infection) was 20. According to the manufacturer’s protocol, puromycin (50 μg/mL) was used to screen for uninfected cells, and the surviving cells were further cultured and expanded.

### Immunofluorescence staining

All specimens were collected from hospitalized patients at the Renmin Hospital of Wuhan University between January 2019 and March 2022. Wax blocks were obtained from the Pathology Department. All patients provided written informed consent. The prepared tissue sections were treated with 3% H_2_O_2_ for 10 min according to routine protocols. After three washes with PBS, the samples were blocked in 3% bovine serum albumin (BSA) and 0.3% Triton X-100 for one h before being incubated with primary antibody overnight at 4° C. After another PBS wash, the samples were incubated with the corresponding fluorescent secondary antibody for one h at 37° C. The nuclei were counterstained with DAPI. Multiplex immunofluorescence staining was performed using TSA fluorescence kits (Servicebio, Wuhan, China) according to the manufacturer’s instructions. Images were acquired using a fluorescence microscope (Olympus BX51). The primary antibodies and dilutions used were as follows: anti-XCL2 (1:200; MAC070Hu22, Cloud-Clone Corp., Wuhan, China), anti-CD68 (1:200; #97778, CST, USA), and anti-iNOS (1:200; #13120, CST).

### Western blot analysis

Briefly, the cell samples were collected and lysed in RIPA buffer (Beyotime) containing 0.1 mM PMSF (Beyotime) and phosphatase inhibitors (Beyotime) for 30 min on ice. The lysate was then centrifuged, and the supernatant was collected. The BCA method (Beyotime) determined the protein concentration. Protein samples were subjected to 12 or 15% sodium dodecyl sulfate-polyacrylamide gel electrophoresis and transferred to PVDF membranes based on molecular weight. The PVDF membranes were blocked with 5% nonfat milk for 1 h at room temperature and washed thrice with TBS-T. The PVDF membranes were incubated with the corresponding primary antibodies overnight at 4° C before being washed three times with TBS-T. Membranes were then incubated with goat anti-mouse IgG (SA00001-1, Proteintech, USA) for 1 h at room temperature. Finally, the bands were scanned, and images were acquired using the ChemiDoc™ Touch Imaging System (Bio-Rad, USA). The results were analyzed using ImageJ software. The experiment was repeated thrice.

### Coculture assay for the migration of M1 macrophage

A 6-well Transwell plate (Corning, USA) was used for the cell migration assays. The XCL2-OE and XCL2-NC groups of 786O, MDA-MB-231, and U251 cells (5 × 10^4^ cells) were added to the lower chamber, and M1 macrophages (5 × 10^4^) were added to the upper chamber. The non-migrated cells in the upper chamber were removed after 24 h of co-culture, and the migrated cells were fixed with 4% paraformaldehyde. Finally, the cells were stained with 0.5% crystal violet solution, and images were captured using an inverted microscope (Olympus, Tokyo, Japan).

### Statistical analysis

Statistical analyses were performed using the R software (version 4.2.1). Spearman’s method was used to evaluate the correlations between certain variables. Student’s t-test or one-way ANOVA test was used using SPSS 19.0 software (SPSS Inc., USA) was used for data comparisons between two or more groups. Graphs were created using the GraphPad Prism software (version 5.0). All data are presented as mean ± standard deviation (SD). Statistical significance was set at P < 0.05.

### Availability of data and materials

All raw data are provided as required.

## RESULTS

### Differential expression of XCL2 in pan-cancer

**Figure 1:**
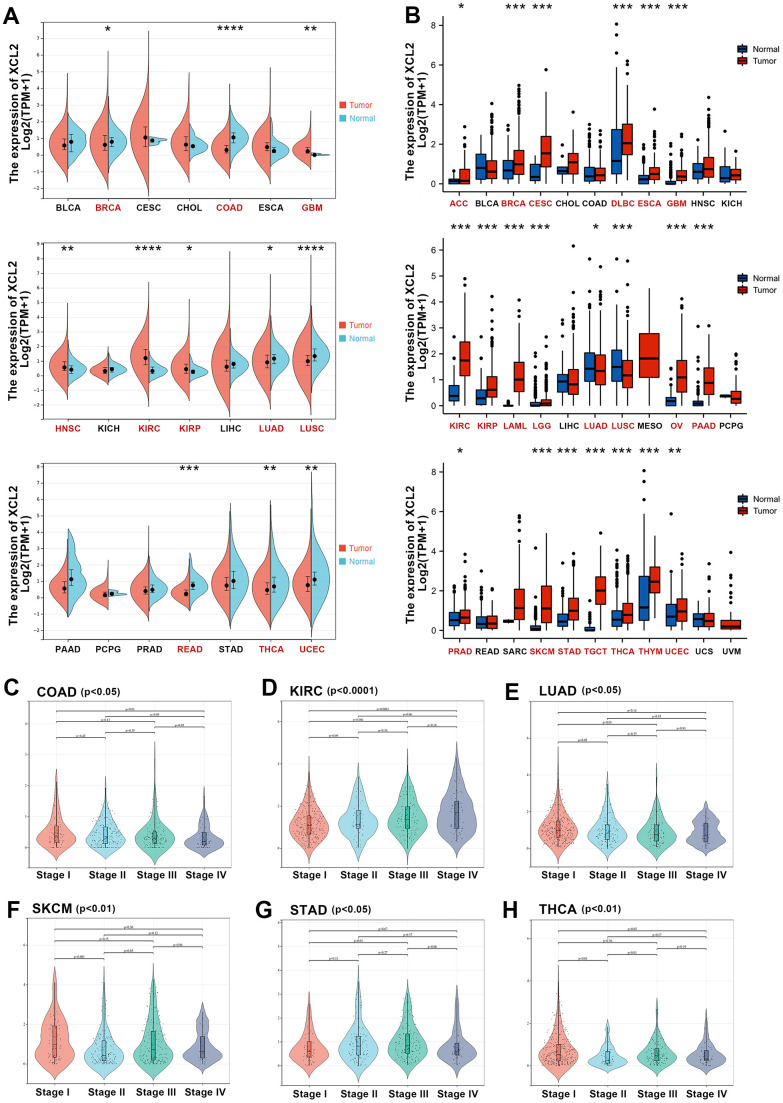
**The expression levels of XCL2 mRNA in pan-cancer.** (**A**) The mRNA expression levels of XCL2 in 21 types of tumors tissues and adjacent normal tissues from the TCGA database; (**B**) Expression of XCL2 between the 33 cancers and normal tissues in unpaired sample analysis; (**C**–**H**) The correlation between the XCL2 expression and cancer stages in COAD, KIRC, LUAD, SKCM, STAD, and THCA (*P < 0.05, **P < 0.01, ***P < 0.001).

First, we investigated the mRNA expression levels of XCL2 in 21 different tumor tissues and adjacent normal tissues from TCGA database (Figure 1A), which indicated that XCL2 expression was significantly different in 11 cancer types, including BRCA, COAD, GBM, HNSC, KIRC, KIRP, LUAD, LUSC, READ, THCA, and UCEC, and that it was significantly lower in most of the cancer tissues than in normal tissues. Additionally, nine cancer types (ACC, LAML, LGG, MESO, OV, SARC, SKCM, TGCT, THYM, UCS, and UVM) were excluded from the expression difference analysis because of the insufficient number of normal tissue samples or the presence of no more than three normal samples. Since the TCGA database primarily contains tumor tissue data and relatively little data from normal tissues, to conduct a more comprehensive differential analysis of XCL2 expression, we also included samples from the GTEx database (15,775 samples), as it also contains RNA-seq data from normal human tissues (Figure 1B). Differences were significant for 21 of the 33 cancers when data from TCGA and GTEx were combined.

Additionally, we used the SangerBox platform to determine whether there was a correlation between XCL2 expression and the pathological stages of various cancers. A significant correlation between different pathological stages and XCL2 expression levels was found in COAD, KIRC, LUAD, SKCM, STAD, and THCA (Figure 1C–1H). Additionally, we found that in many cancers, with an increase in pathological stage, XCL2 expression decreased.

### Prognostic analysis of XCL2 in the 33 cancers

**Figure 2:**
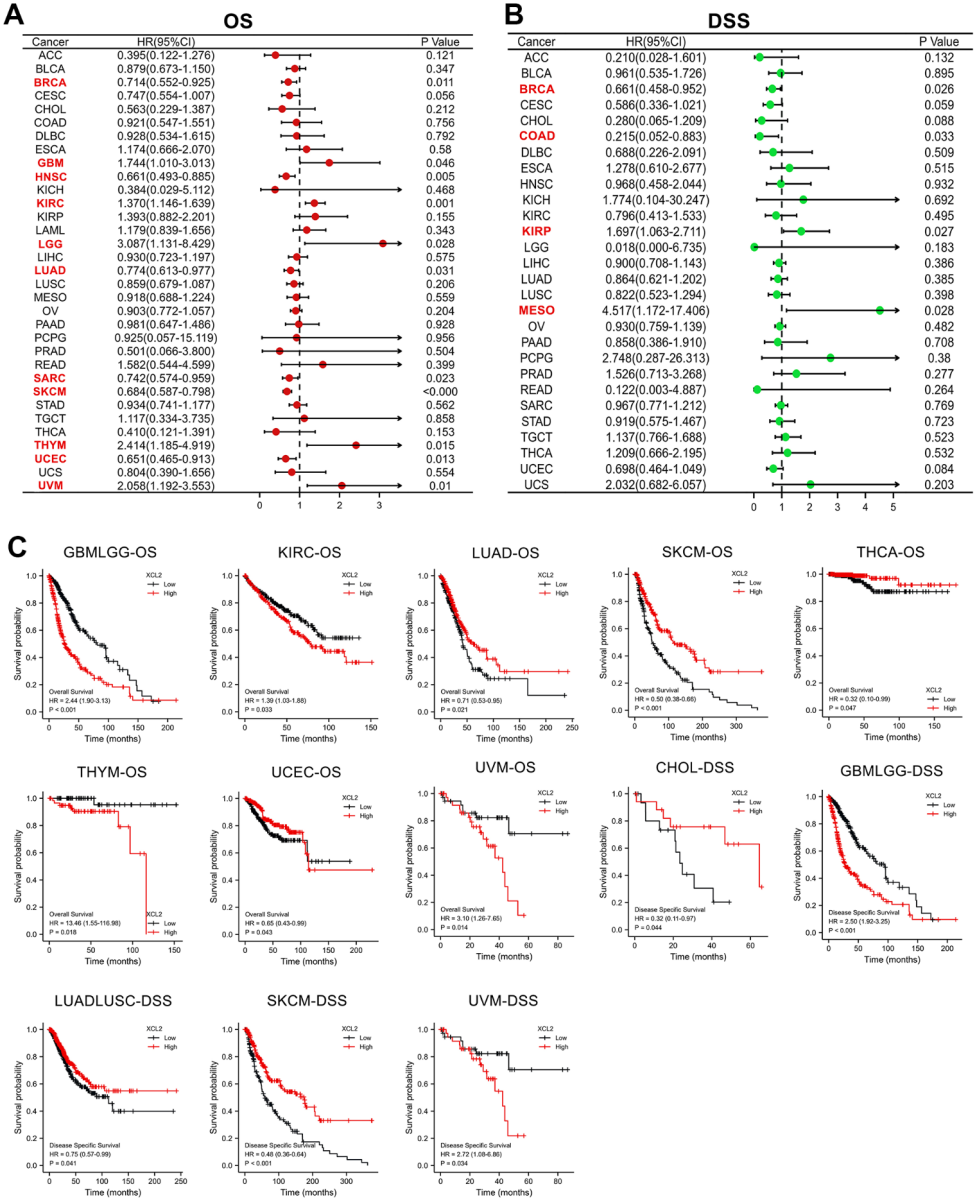
**The univariate Cox regression and Kaplan–Meier survival analyses of XCL2 in pan-cancer.** (**A**) The relationship between XCL2 expression levels and OS in various cancer types through single variate Cox regression analysis using TCGA database; (**B**) The relationship between XCL2 expression levels and DSS in various cancer types through single variate Cox regression analysis using TCGA database; (**C**) Kaplan–Meier analysis of the association between XCL2 expression and OS/DSS.

Furthermore, we analyzed the prognostic effect of XCL2 in 33 cancer types using univariate Cox regression analyses and Kaplan–Meier survival curves. Univariate Cox regression analysis of OS (Figure 2A) revealed that high XCL2 levels were significant risk factors for BRCA (P = 0.011), GBM (P = 0.046), HNSC (P = 0.005), KIRC (P = 0.001), LGG (P = 0.028), LUAD (P = 0.031), SARC (P = 0.023), SKCM (P < 0.001), THYM (P = 0.015), UCEC (P = 0.013), and UVM (P = 0.01). As for DFS (Figure 2B), the univariate analysis showed that high expression of XCL2 was strongly correlated with poor DSS in BRCA (P = 0.026), COAD (P = 0.033), KIRP (P = 0.027), and MESO (P = 0.028).

Kaplan–Meier survival curves demonstrated that increased XCL2 expression was significantly associated with worse OS in GBMLGG (P < 0.001), KIRC (P = 0.033), THYM (P = 0.018), and UVM (P = 0.014). At the same time, the results were reversed for LUAD (P = 0.021), SKCM (P < 0.001), LUAD (P = 0.021), THCA (P = 0.047), and UCEC (P = 0.043). Meanwhile, DSS results displayed that XCL2 level was linked to the patient’s better prognosis in CHOL (P = 0.044) and LUADLUSC (P = 0.041) and worse prognosis in GBMLGG (P < 0.001) and UVM (P = 0.034) (Figure 2C). The results indicate that XCL2 is most correlated with prognosis in SKCM, and high expression of XCL2 can delay the progression of SKCM.

### XCL2 mutation and methylation profile in pan-cancer based on TCGA

**Figure 3:**
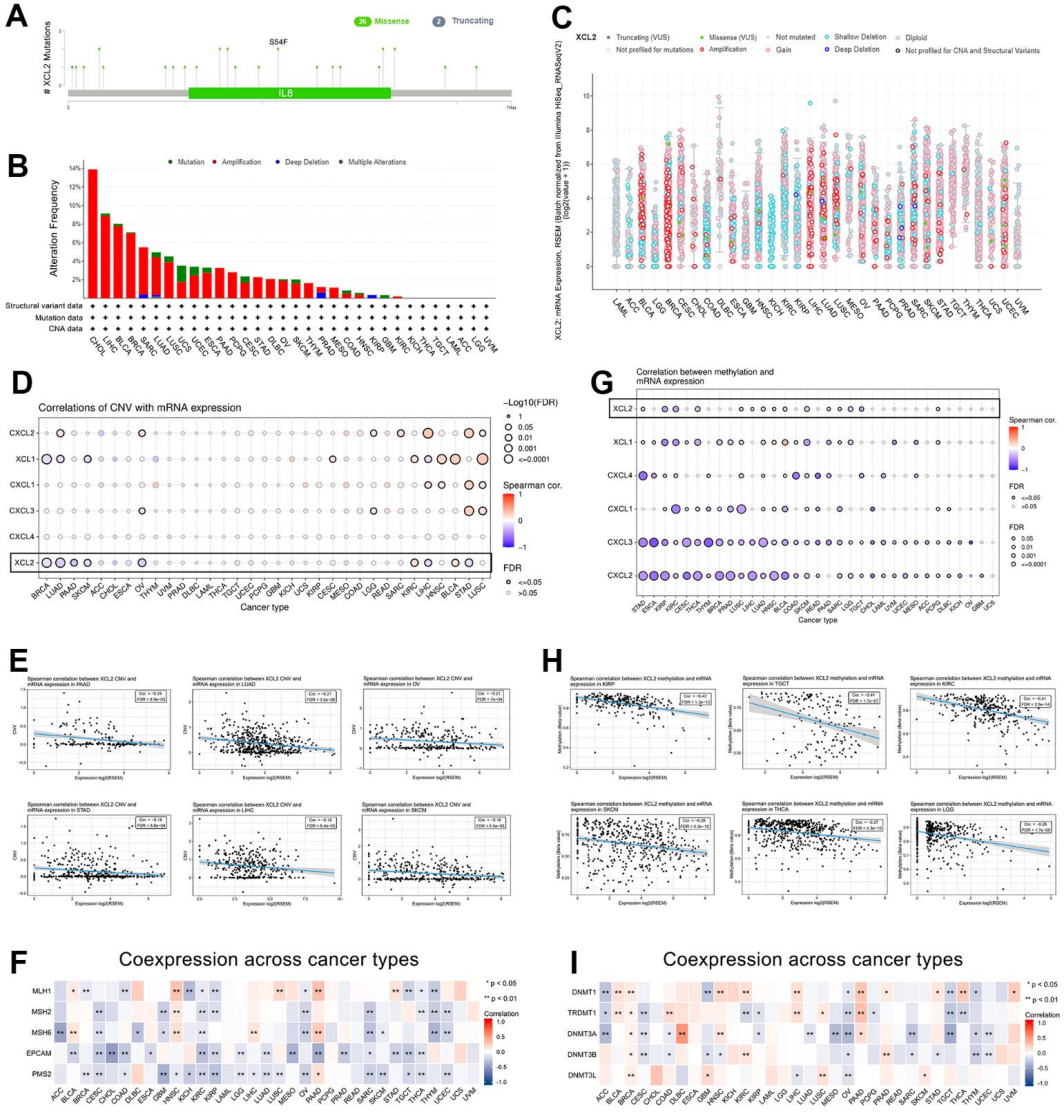
**The gene mutation character of XCL2 in pan-cancer.** (**A**) The mutation sites, types of mutation, and alteration frequency of XCL2 somatic mutation; (**B**, **C**) The mutation frequency of XCL2 in various cancer using the cBioPortal database; (**D**, **E**) The correlation of CNV with XCL2 mRNA expression and 5 XCL-related genes in 33 cancers; (**F**) The co-expression between MMR-related genes and XCL2; (**G**, **H**) The correlation of methylation with mRNA expression of XCL2 and 5 XCL-related genes in 33 cancers; (**I**) The co-expression between methylation-related genes and XCL2 (*P < 0.05, **P < 0.01, ***P < 0.001).

Individuals with cancer typically have highly heterogeneous somatic mutations in multiple genes [21–23]. Consequently, gene mutations are closely linked to cancer development, and the forms of such mutations are variable. The GSCA and cBioPortal were utilized to analyze the distribution of mutation sites, types of mutation, and alteration frequency. Figure 3A presents the sites between amino acids 0–114 and the number of XCL2 mutations. These results demonstrate that XCL2 mutations appear to be conserved in tumorigenesis. However, as a form of gene alteration, amplification of XCL2 was more common in pan-cancer than in mutations, with the highest amplification rate in CHOL (14%) (Figure 3B, 3C). Furthermore, we found that the SNV rate of XCL2 was 23% in 100 samples, consisting of splice sites, missense mutations, and nonsense mutations (Supplementary Figure 1A). Heterozygous amplifications and deletions of XCL2 were common in the 33 cancer types (Supplementary Figure 1B). Additionally, XCL1 and XCL2 showed significant homologous amplification in BLCA, BRCA, CHOL, LIHC, LUAD, LUSC, and SARC but not in CXCL1, CXCL2, CXCL3, and CXCL4 (Supplementary Figure 1C). We also found that increased XCL2mRNA expression was significantly associated with CNV (Figure 3D, 3E). MMR-related genes were negatively correlated with XCL2 expression in most cancers (Figure 3F).

Methylation is a widely studied epigenetic mechanism that plays a crucial role in the development and progression of cancer [24, 25]. Therefore, we explored the correlation between XCL2 methylation and mRNA expression and found that XCL2 expression was negatively associated with methylation levels in some cancers (Figure 3G–3I).

### XCL2 expression affected immune checkpoint genes, TMB, and MSI

**Figure 4:**
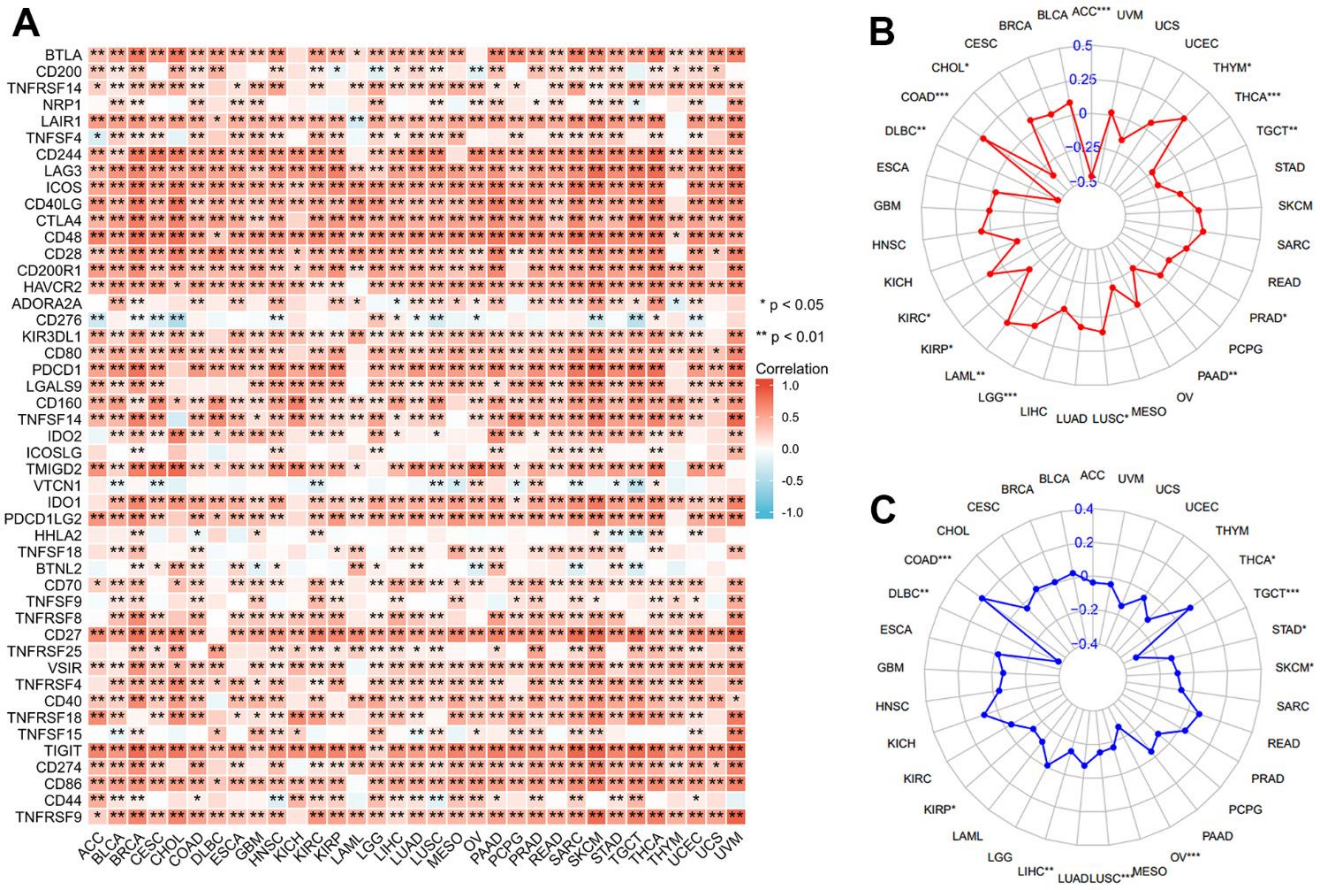
**Correlation of XCL2 with immune checkpoint genes, TMB, and MSI.** Correlation between XCL2 expression and immune checkpoint genes (**A**), TMB (**B**), and MSI (**C**) (*P < 0.05, **P < 0.01, ***P < 0.001).

The critical role of immune checkpoint-related genes has been well-studied in immunotherapy since its development [26, 27]. A strong positive correlation was found between 47 known immune checkpoint genes and XCL2 expression in 33 cancers (Figure 4A). We also observed positive associations between XCL2 and TMB in COAD, KIRC, LAML, LGG, LUSC, and THYM and negative associations in ACC, CHOL, DLBC, KIRP, PAAD, PRAD, TGCT, and THCA (Figure 4B). For MSI, DLBC, KIRP, LIHC, LUSC, OV, SKCM, STAD, and TGCT were negatively correlated, whereas COAD and THCA demonstrated positive correlations (Figure 4C).

### XCL2 is strongly relevant to immune infiltration

**Figure 5:**
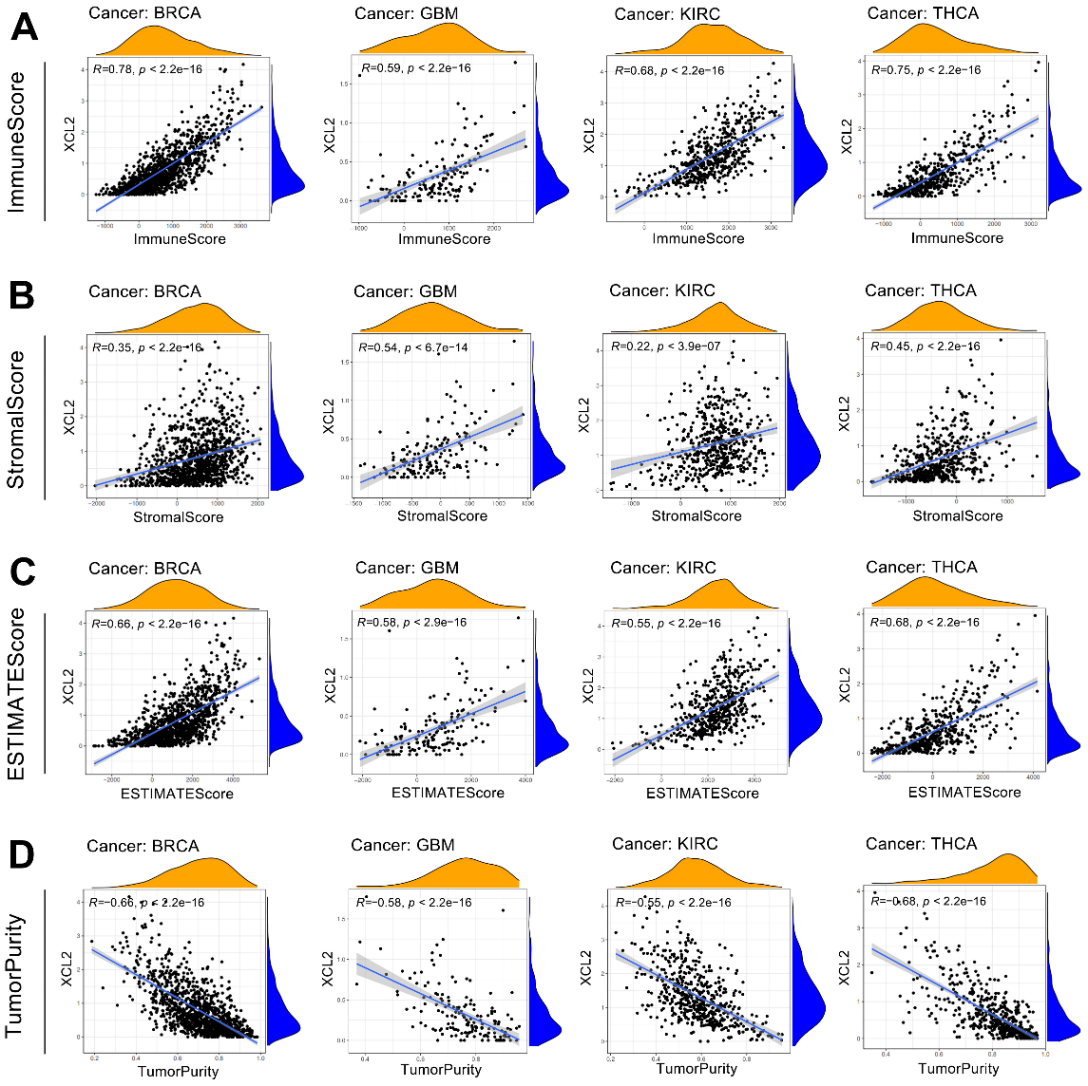
Analysis of XCL2 and the tumor microenvironment in four cancer types with top correlation coefficients, including BRCA, GBM, KIRC, and THCA (**A**–**D**).

The presence of immune cells in the immune microenvironment can be a potential target for immunotherapy, and XCL2 is a chemokine that recruits many immune cells [16]. Therefore, we investigated the relationship between XCL2 expression and the degree of immune cell infiltration to examine the role of XCL2 in tumor immunity. We calculated the stromal, immune, and ESTIMATE scores and tumor purity of 33 cancers using Spearman’s correlation analysis and found that immune scores were positively associated with XCL2 expression, especially in BRCA, GBM, KIRC, and THCA (Figure 5A–5D).

**Figure 6:**
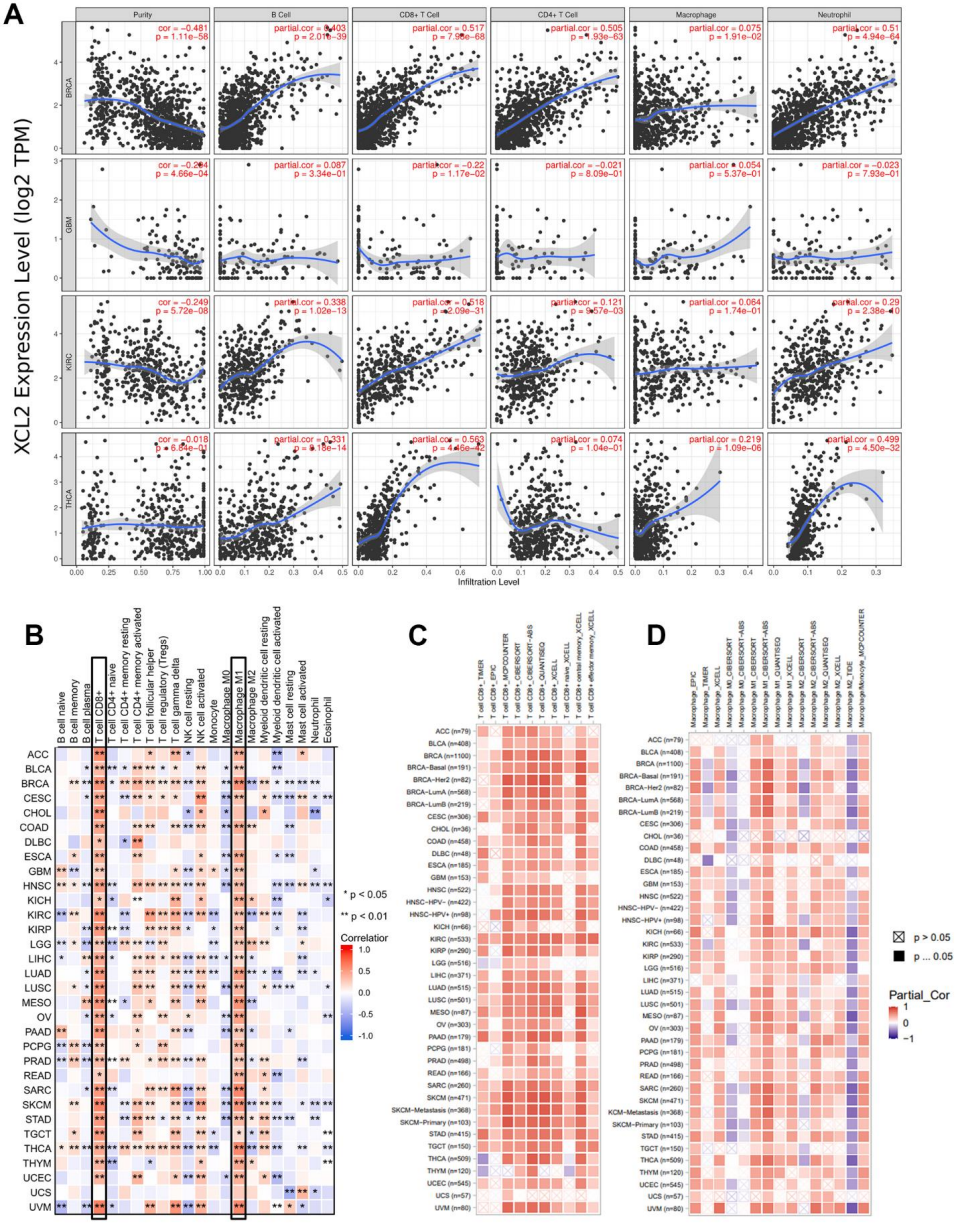
**Analysis between the XCL2 expression and immune cell infiltration.** (**A**) TIMER2.0 database was used to exhibit the correlations between XCL2 expression and immune cells (B, CD4+ T, CD8+ T, macrophages, neutrophils); (**B**) Positive correlation between CD8 T cells and macrophages M1 and XCL2 expression in pan-cancer under the CIBERSORT calculation method; (**C**) CD8 T cells show a strong positive correlation with XCL2 expression in the majority of cancers; (**D**) Correlation between various subtypes of macrophages and XCL2 expression under different algorithms (*P < 0.05, **P < 0.01, ***P < 0.001).

Using TIMER2.0, we also found that XCL2 expression was positively or negatively correlated with B, CD4+ T, CD8+ T, and neutrophils in BRCA, GBM, KIRC, and THCA, but the correlation was weakest in macrophages (Figure 6A). Subsequently, we independently extracted the immune cell infiltration of the tumor microenvironment calculated by the CIBERSORGT algorithm. We demonstrated that high expression of XCL2 correlated strongly and positively with M1 macrophages and CD8 T cells in pan-cancer, whereas other cells (e.g., macrophage type M0, macrophage type M2, and CD4 T cells) showed inconsistent correlations (Figure 6B). We then calculated the correlation between the expression of CD8 + T cells, macrophages, and XCL2 in 33 cancers. We found a positive correlation between XCL2 expression and CD8 ^+^ T cells and macrophages (especially M1 macrophages) in pan-cancer (Figure 6C, 6D). This is consistent with our finding that XCL2 expression levels positively correlate with the infiltration of M1 macrophages and T lymphocytes.

### Sing-cell RNA sequencing (scRNA-seq) analysis of XCL2

**Figure 7:**
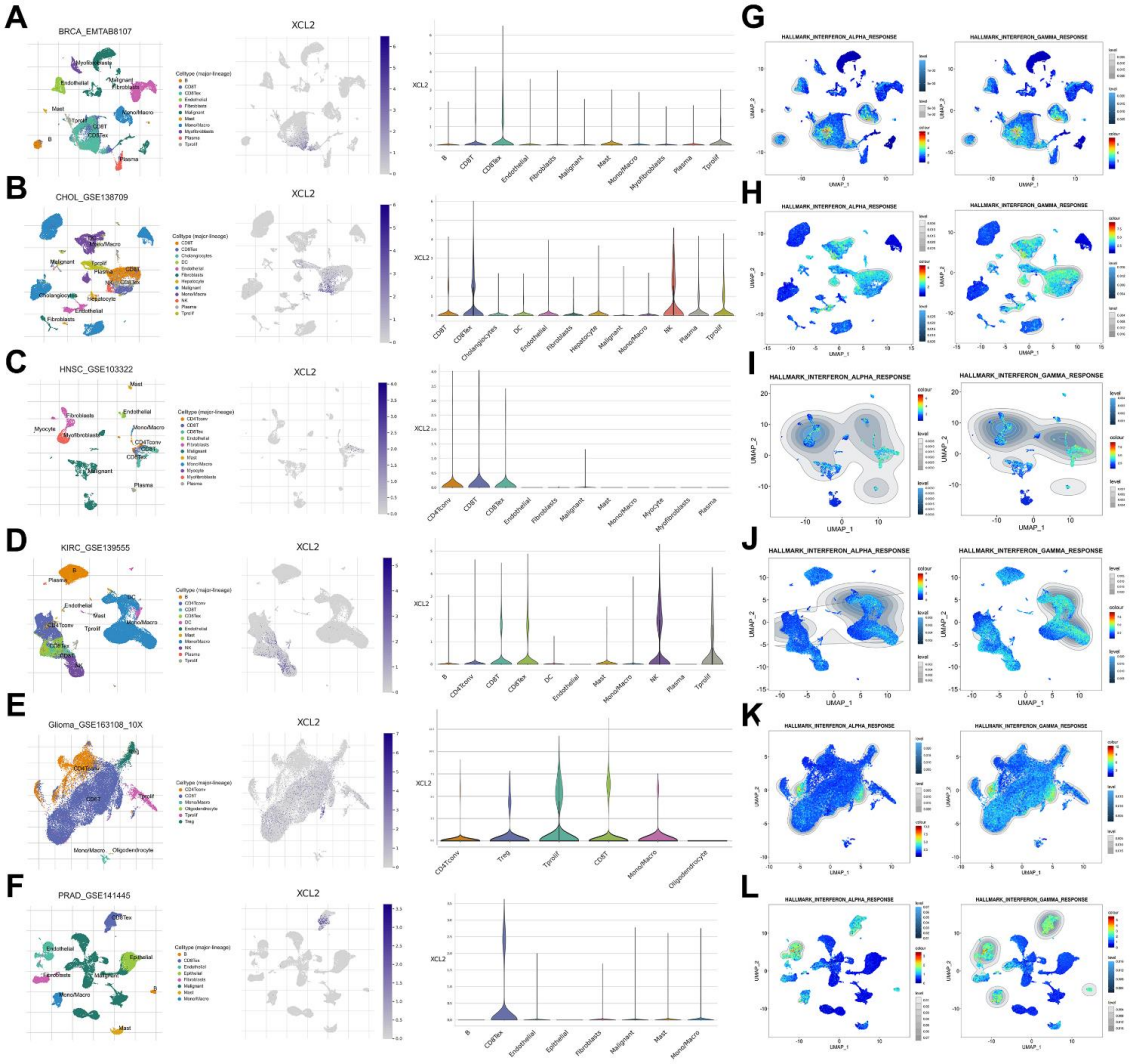
**The scRNA-seq results of XCL2 expression and single-cell signature in pan-cancers.** The definition of cancer cells in BRCA (**A**), CHOL (**B**), HNSC (**C**), KIRC (**D**), GBM (**E**), and PRAD (**F**). Enrichment analyses of interferon-alpha and interferon-gamma responses in BRCA (**G**), CHOL (**H**), HNSC (**I**), KIRC (**J**), GBM (**K**) and PRAD (**L**) single cell sequencing results.

We used scRNA-seq to delineate the expression levels of XCL2 and its immune infiltrative role in BRCA, CHOL, HNSC, LAML, KIRC, KICH, GBM, OV, LIHC, PRAD, and THCA. Different cell surface markers distinguished different clusters of infiltrating immune cell types (e.g., monocytes, B cells, CD8T cells, CD8Tex, and NK cells) in each tumor sample. The expression of XCL2 in different cell clusters in each tumor sample is displayed in Figure 7 and Supplementary Figure 2. The trend of XCL2 expression in cell clusters was nearly identical in all tumor samples. For example, XCL2 is widely distributed in cancer and various immune cells. However, we identified abundant expression in all types of CD8+ T cells in the cancers mentioned above, whereas NK cells showed high XCL2 expression in CHOL and KIRC (Figure 7 and Supplementary Figure 2). Notably, XCL2 was also highly expressed in macrophages from BRCA, GBM, PRAD, and KIRC (Figure 7A, 7G, 7I, 7K). Additionally, we found that interferon-alpha and interferon-gamma responses were primarily enriched in CD8+ T cells and macrophages in almost all cancers by exploring single-cell characteristics (Figure 7 and Supplementary Figure 2).

### XCL2 mediates tumor cell invasion and M1 macrophage migration *in vivo*

**Figure 8:**
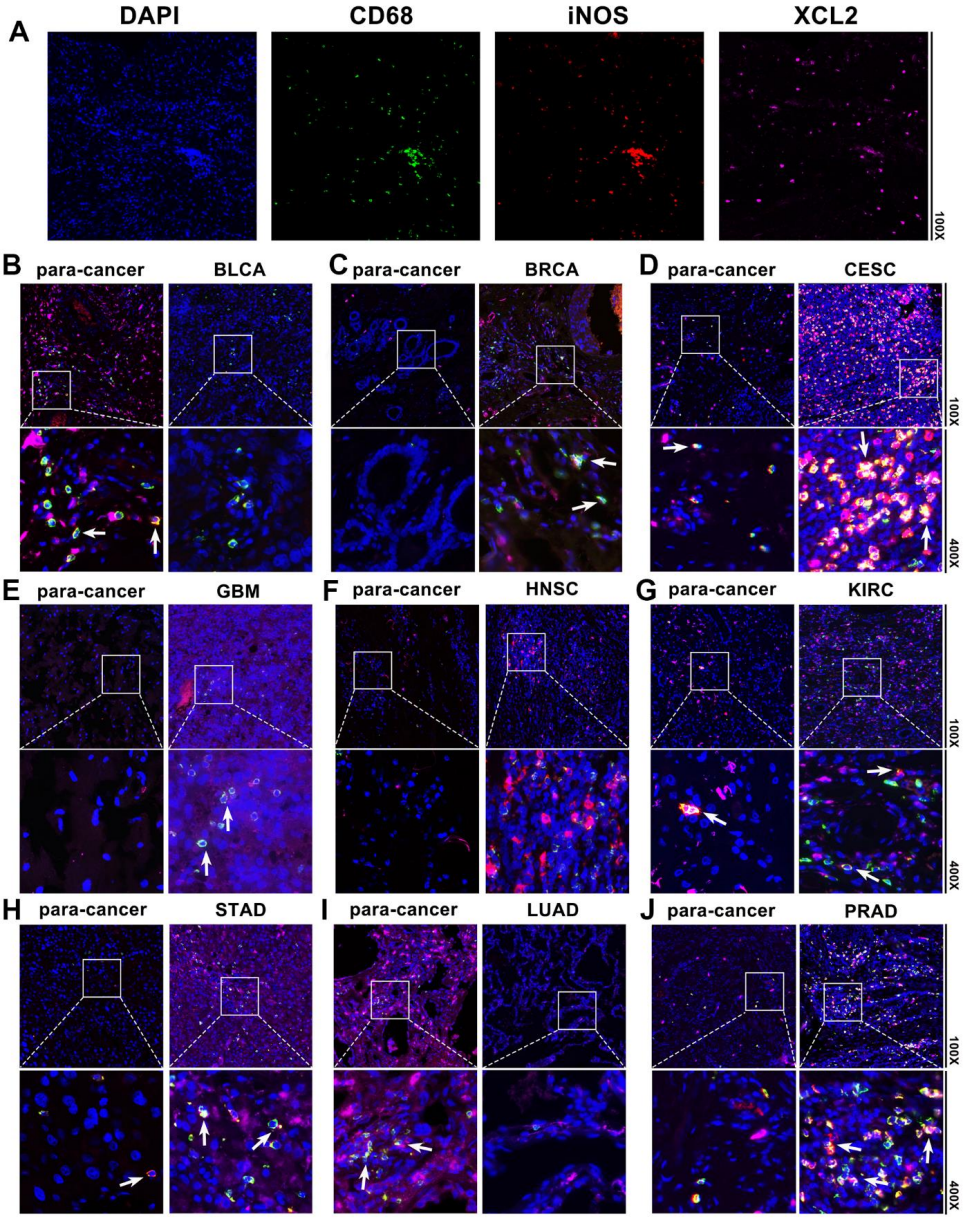
**Pan-cancer samples and the associated para-cancerous tissues were stained using multiplex immunofluorescence.** The typical picture of the staining for DAPI, CD68, iNOS, and XCL2 in pan-cancer samples is shown in (**A**). Red, green, and pink represent CD68-, iNOS-, and XCL2-positive cells, respectively. Blue denotes the DAPI-stained nucleus. (**B**) Bladder urothelial carcinoma (BLCA), (**C**) invasive breast carcinoma (BRCA), (**D**) cervical squamous cell carcinoma and endocervical adenocarcinoma (CESC), (**E**) glioblastoma multiforme (GBM), (**F**) head and neck squamous cell carcinoma (HNSC), (**G**) kidney renal clear cell carcinoma (KIRC), (**H**) stomach adenocarcinoma (STAD), (**I**) lung adenocarcinoma (LUAD), and (**J**) Prostate adenocarcinoma (PRAD). XCL2 and iNOS double-positive cells with 100× and 400× amplification are shown by the note.

We also detected XCL2 expression in BLCA, BRCA, CESC, GBM, HNSC, KIRC, STAD, LUAD, and PRAD using pan-cancer samples. According to Figure 8, immunofluorescence labeling revealed that BLCA, BRCA, CESC, GBM, HNSC, KIRC, STAD, LUAD, and PRAD had higher numbers of XCL2-positive cells as compared to the equivalent para-cancerous tissues. XCL2 expression levels increase as the disease progresses in various cancers. Additionally, most pan-cancer samples had a much higher proportion of M1 macrophage marker iNOS-positive cells than para-cancerous tissues (Figure 8), indicating the potential involvement of XCL2 in the recruitment of M1 macrophages in the TME.

### XCL2 mediates M1 macrophage migration *in vitro*

**Figure 9:**
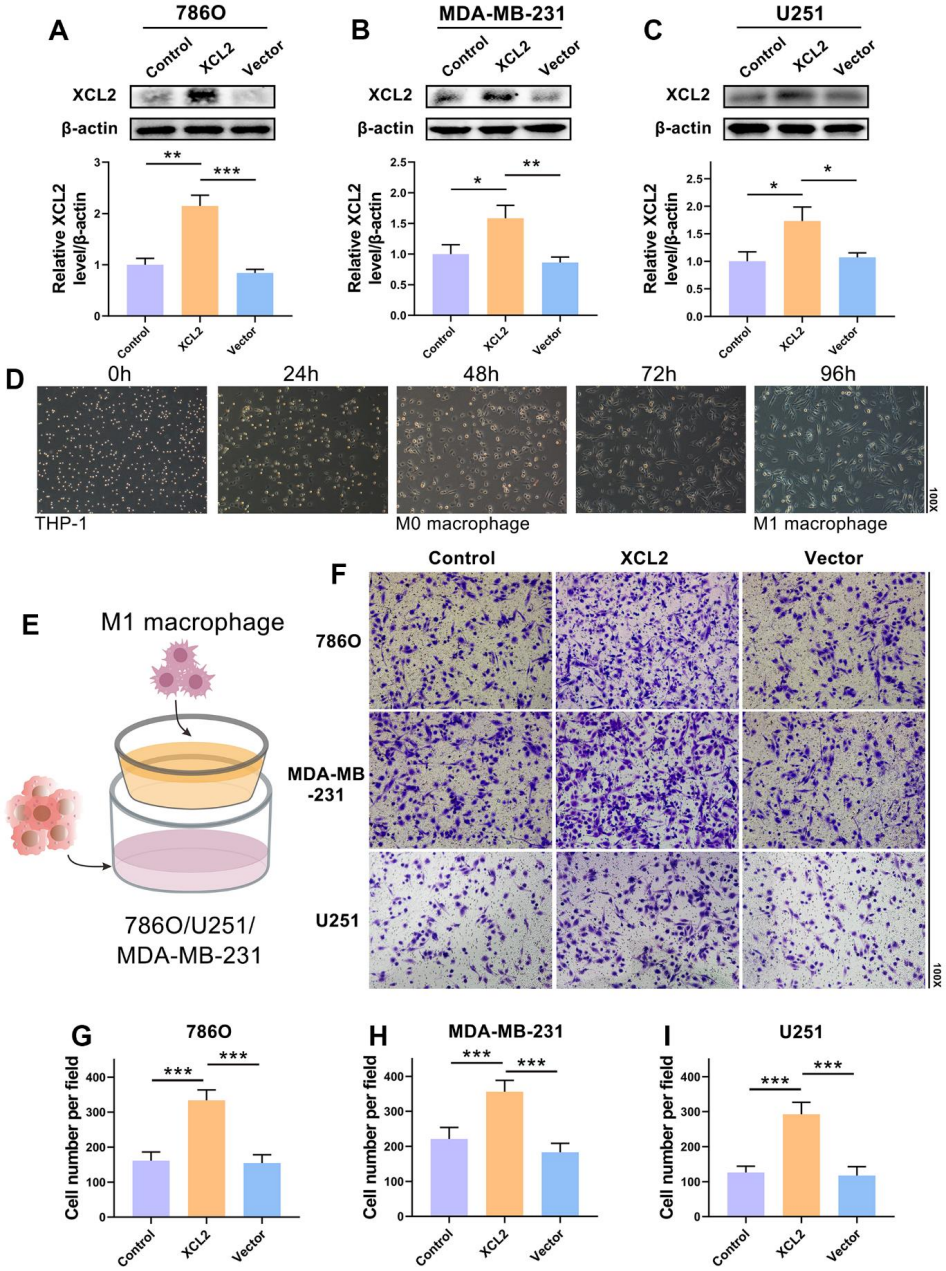
**M1 macrophage migration is mediated by XCL2.** (**A**–**C**) The transduction outcomes of XCL2 in 786O, MDA-MB-231, and U251 are confirmed using western blotting. (**D**) The M1 macrophages induction procedure and morphology. (**E**) The schematic design shows the coculture of 786O, MDA-MB-231, or U251 cells with M1 macrophages. (**F**–**I**) The movement of M1 macrophages in coculture with 786O, MDA-MB-231, and U251 cells that have been XCL2-OE and XCL-NC transfected. The magnification under the microscope is shown as marked in the figure. *P < 0.05, **P < 0.01, and ***P < 0.001.

The human KIRC cell line 786O, GBM cell line U251, and BRCA cell line MDA-MB-231 were transfected with a lentivirus. According to the western blotting findings, XCL2 significantly increased the protein level of XCL2 compared to the vector (Figure 9A–9C). The ability of M1 macrophages to migrate was further examined to determine whether XCL2 suppression had any effect on 786O, MDA-MB-231, and U251 cells. Human THP-1 cells were induced to differentiate into M1 macrophages (Figure 9D). We then co-cultured M1 macrophages with 786O, MDA-MB-231, and U251 cells using a transwell apparatus (Figure 9E). Similarly, overexpression of XCL2 in 786O, MDA-MB-231, and U251 cells enhanced the ability of M1 macrophages to migrate when co-cultured (Figure 9F–9I). Our results showed that XCL2 may mediate M1 macrophage migration.

### Significant differences in XCL2 expression in different immune and molecular subtypes and showed potential therapeutic value in immunotherapy response

Studies have shown that elucidating tumor and immune cell interplay would assist in predicting immunotherapy responses and developing novel immunotherapy targets [28, 29]. Previous survival studies have found that XCL2 may be associated with the prognosis of 15 cancers. The results showed significant XCL2 expression in various immune subtypes in 13 out of 16 cancers, including ACC (six subtypes), BRCA (five subtypes), CESC (three subtypes), HNSC (five subtypes), KIRC (six subtypes), KIRP (six subtypes), LUAD (five subtypes), SARC (five subtypes), SKCM (five subtypes), STAD (five subtypes), THCA (five subtypes), UCEC (five subtypes), and UVM (three subtypes) (Supplementary Figure 3A–3M).

**Figure 10:**
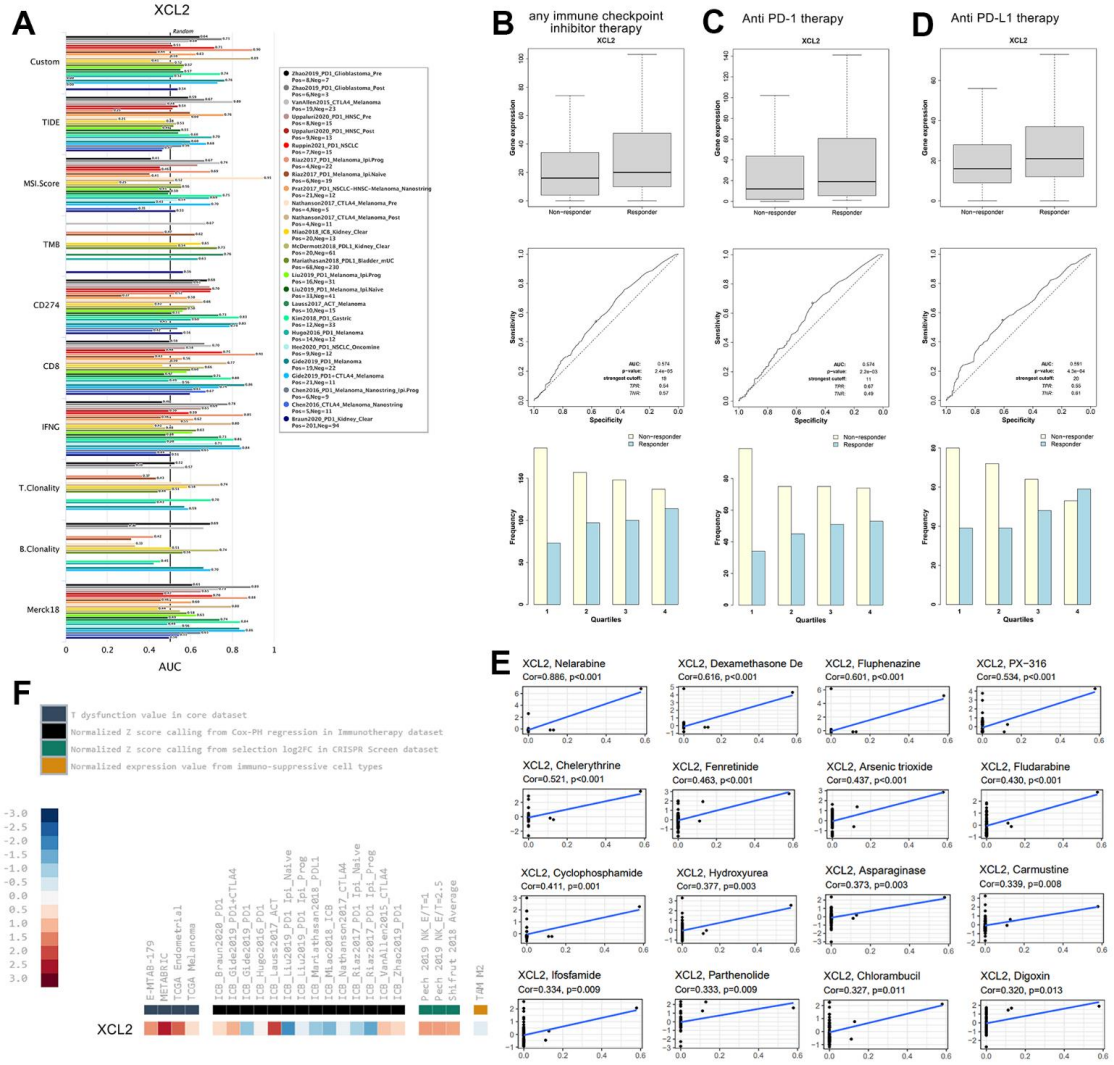
**XCL2 showed potential therapeutic value in immunotherapy response** (**A**) We performed the comparison between XCL2 and other published biomarkers based on response outcomes and the predictive power of overall survival; (**B**) Expression of XCL2 varies across patients with different responsiveness throughout immune checkpoint inhibitor therapy; (**C**) Expression of XCL2 varies in patients with different responsiveness in anti PD-1 treatment; (**D**) Expression of XCL2 varies in patients with different responsiveness in anti PD-L1 treatment; (**E**) The relationship between CDCA4 expression and expected medication response; (**F**) The expression levels of XCL2 in different datasets.

We estimated multiple published transcriptomic biomarkers based on the expression profile before tumor treatment and used this information to predict patient responses. The TIDE platform was used to compare XCL2 expression with other published biomarkers regarding response outcomes and the predictive power of overall survival, the results of which are revealed in Figure 10A. We found that the AUC for XCL2 was greater than 0.5 in 18 of the 25 immunotherapy cohorts, whereas the corresponding values for MSI Score (AUC > 0.5 in 13 immunotherapy cohorts), TMB (AUC > 0.5 in eight immunotherapy cohorts), IFNG (AUC > 0.5, in 17 immunotherapy cohorts), T. Clonality (AUC > 0.5, in nine immunotherapy cohorts), and B. Clonality (AUC > 0.5, in seven immunotherapy cohorts). However, the predictive value of XCL2 was lower than that of CD274, which had an AUC value greater than 0.5 in 21 immunotherapy cohorts and had the same predictive value as TIDE, CD8, and Merck18, all with AUC values greater than 0.5 in 18 immunotherapy cohorts. Further studies on the response to immune checkpoint inhibitor therapy have shown that responders exhibited higher XCL2 expression than non-responders in pan-cancer (Figure 10B). Patients with high XCL2 expression levels were more sensitive to treatment (Figures 10C, 10D). We found a significant increase in responders and a significant decrease in non-responders from the first to the fourth groups by quartile differentiation (Chi-square analysis was performed in this study, P < 0.001). XCL2 expression levels were upregulated in core and CRISPR screening datasets. However, XCL2 expression was upregulated and downregulated in the other immunotherapy databases (Figure 10F).

Meanwhile, analysis of data from the Cellminer database showed that CDCA4 expression was positively correlated with drug response after receiving medications such as Nelarabine, Dexamethasone De, Fluphenazine, PX-316, Chelerythrine, Fenretinide, Arsenic trioxide, Fludarabine, Cyclophosphamide, Hydroxyurea, Asparaginase, Carmustine, Ifosfamide, Parthenolide, Chlorambucil, and Digoxin (Figure 10E). These findings demonstrate that XCL2 has predictive value for immunotherapeutic response in human cancer.

### Construction of the protein-protein interaction network, functional enrichment and gene set enrichment

**Figure 11:**
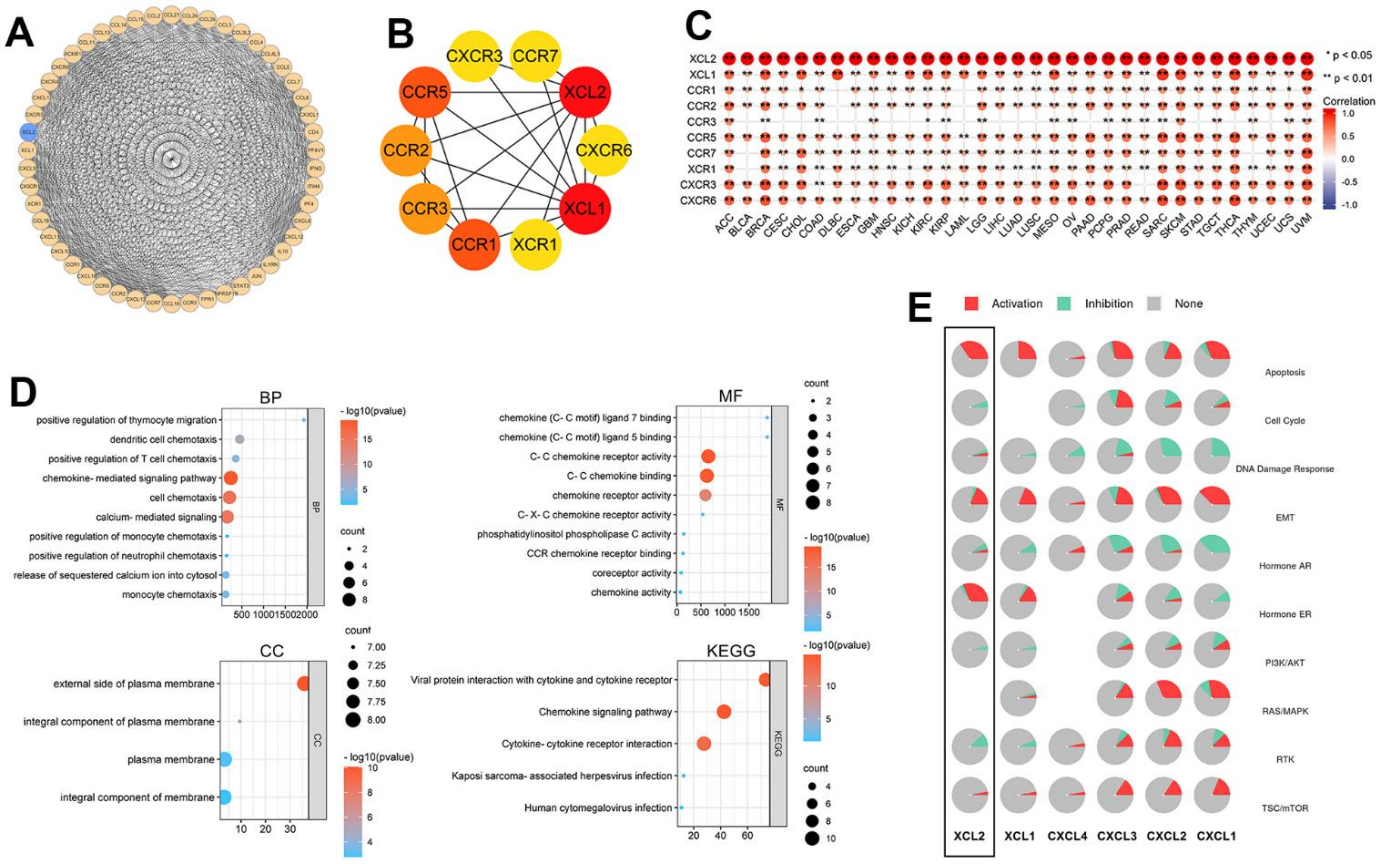
**The PPI network and gene set enrichment of XCL2.** (**A**) The top 50 genes related to XCL2 were constructed to a PPI network; (**B**) The top 10 genes of the PPI network; (**C**) The correlation of the top 10 hub genes in 33 cancers (*P < 0.05, **P < 0.01, ***P < 0.001); (**D**) GO and KEGG enrichment analysis of top 10 hub genes in PPI network; (**E**) Activation or inhibition of common signaling pathways by XCL2.

To further explore the biological function of XCL2, the STRING database was used to identify the 50 genes most closely associated with XCL2 and a PPI network was built using Cytoscape (Figure 11A). Using the MCC cytoHubba ranking, we derived the 10 most relevant genes in the PPI network: XCL1, CCR1, CCR5, CCR2, CCR3, XCR1, CXCR6, CXCR3, and CCR7 (Figure 11B). The top 10 genes closely related to 33 cancers were mainly chemokines and played similar roles *in vivo*, as shown in Figure 11C. We then performed a GO/KEGG enrichment analysis using these genes (Figure 11D). The biological process (BP) enriched in this dataset was most relevant to the positive regulation of thymocyte migration, dendritic cell chemotaxis, and the positive regulation of T cell chemotaxis. In contrast, cellular components (CC) were associated with some biological functions of the plasma membrane, including the external side of the plasma membrane and an integral component of the plasma membrane. The top GO terms of molecular function (MF) were chemokine ligand 7/5 binding and C-C chemokine receptor activity. The top three KEGG pathways were viral protein interaction with cytokine and cytokine receptors, chemokine signaling pathways, and cytokine-cytokine receptor interactions. Overexpression of XCL2 can significantly activate apoptosis, EMT, Hormone ER, and TSC/mTOR signaling pathways and inhibit the cell cycle, PI3K/AKT, and RTK pathways (Figure 11E).

**Figure 12:**
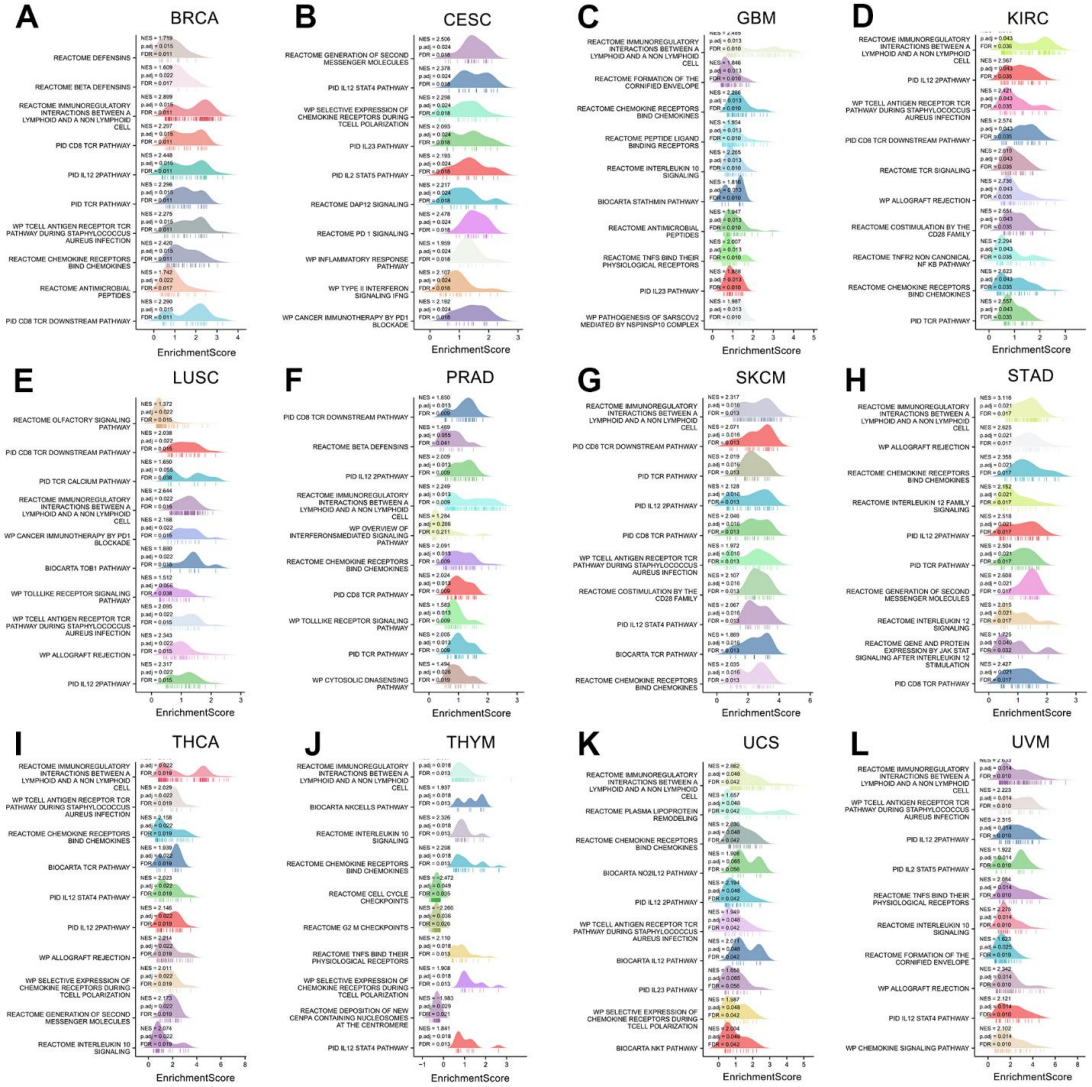
**The gene set enrichment analysis of XCL2 in 12 cancers.** The top 10 GSEA pathways of XCL2 in (**A**) BRCA, (**B**) CESC, (**C**) GBM, (**D**) KIRC, (**E**) LUSC, (**F**) PRAD, (**G**) SKCM, (**H**) STAD, (**I**) THCA, (**J**) THYM, (**K**) UCS, and (**L**) UVM. The Y-axis represents one gene set, and the X-axis is the distribution of enrichment corresponding to the core molecules in each gene set.

We also performed Gene Set Enrichment Analyses (GSEA) on 12 cancers with differential prognoses (Figure 12A–12L). The results of 12 cancers showed that XCL2 is primarily associated with the biological function of T lymphocytes, including T cell polarization, PD-1 blockade, differentiation, chemotaxis, and multiple pathways of T cell-associated inflammatory factors such as IL12, IL23, STAT4, CD8, and TCR pathways. These results suggested that XCL2 is closely related to the processes of multiple lymphocytes, especially T lymphocytes and NK cells, in various cancers.

## DISCUSSION

XCL2 is located on chromosome 1q24.2 and spans approximately 3.23 kb pairs [30]. It is an important chemokine in inflammatory and immune responses. The pathways related to this are the GPCR downstream signal and the integrin pathway. In this study, we comprehensively explored the role of XCL2 in different tumor types by performing panoramic analysis, which is the first of its kind in pan-cancer analysis, using bioinformatics analysis combined with tumor tissue immunofluorescence and macrophage chemotaxis assays. The results showed that XCL2 expression was associated with pan-cancer survival, alterations, immune infiltration, immune subtypes, and drug sensitivity. In conclusion, our findings suggest that XCL2 is a potential target for cancer immunotherapy.

Like many other oncogenes, XCL2 acts as a key molecule in pan-cancer. Several studies [16] demonstrated that XCL2 expression increases with lung cancer progression. In this study, we explored the expression levels of XCL2 mRNA in human organs and tissues and compared them with those observed in various cancers. The results demonstrated that XCL2 acts as a specific oncogene in most tumor types and as an oncogene repressor in tumors and that XCL2 expression is crucial for tumor prognosis. The expression of XCL2 is downregulated in four types of cancers and upregulated in seven types of cancers in TCGA database compared to normal tissues Meanwhile, the K-M plot showed that XCL2 expression is related to prognosis in human cancers, which is consistent with previous analyses of most cancer types. These results provide a foundation for future research.

Previous studies have demonstrated that CNVs and DNA methylation can reduce mRNA levels [31, 32], which correlates closely with patient prognosis. We also systematically analyzed the relationship between XCL2, DNA methylation, and CNVs. The mutation types of XCL2 were mainly substitutions in pan-cancer, and the overall frequency of XCL2 mutations in pan-cancer was low, only approximately 2% in the highest UCS. A few missense mutation sites in XCL2 were detected using the cBioPortal database, indicating a conserved pan-cancer sequence of XCL2. The highest number of samples having mRNA expression in PRAD was found among all samples with deep deletions in XCL2. There is growing evidence of an association between DNA methylation and altered gene expression in cancer [32, 33]. DNA methylation reduced XCL2 mRNA expression and was statistically correlated with KIRP, KIRC, THCA, SKCM, LGG, and TGCT, suggesting that DNA methylation of the XCL2 gene may determine the mRNA levels of XCL2 in cancers. Other protein data have indicated that DNA methylation and mutation frequency influence enzymatic activity, disease progression, and patient prognosis. Nonetheless, evidence for XCL2 remains largely unknown. While the overall rate of XCL2 mutation is high in human cancers, CNVs are inconsistently expressed at the mRNA level in pan-cancers (most have a negative correlation). However, CNVs are caused by the action of XCL2, which is highly expressed in tumor tissues. However, the roles of DNA methylation and XCL2 mutations in cancer development and progression require further investigation.

The effectiveness of tumor immunotherapy requires the presence of sufficient and effective immune cells in the tumor microenvironment to activate immune cells and prevent their immune escape of tumor cells. Several studies [34–36] have confirmed that the type and abundance of T cells and macrophage infiltration profoundly affect tumor progression and prognosis. Effector T cells in the tumor microenvironment (TME) exhibit high levels of expression of multiple inhibitory receptors, such as PD-1, T cell immunoglobulin and mucin-containing protein 3 (TIM3), T cell immune receptors with Ig and ITIM structural domains (TIGIT), and LAG3 [37] (lymphocyte activation 3), which are considered markers of a dysfunctional state widely known as T-cell depletion [38]. Specifically, exhausted T cell populations in non-small cell lung cancer (NSCLC) and hepatocellular carcinoma (HCC) have been identified as a result of increased expression of cells that suppress receptors, thereby promoting immune escape [39, 40]. The present study found that XCL2 expression was positively associated with CD8 + T lymphocytes and M1 macrophages in almost all cancers. Single-cell sequencing results showed that XCL2 was predominantly enriched in CD8+ T exhaustive cells (CD8Tex), suggesting that XCL2 expression promotes dysfunctional CD8+ T cells. This confirmed our earlier suspicion that XCL2 acts as an important chemokine in the recruitment of large numbers of T lymphocytes and macrophages to kill tumor cells. These results were also demonstrated in subsequent *in vivo* and *in vitro* experiments. However, the complex diversity of CD8 + T lymphocytes was not further investigated in this study.

TME, an important condition for tumor development *in vivo*, consists of malignant cells, endothelial cells, fibroblasts, stromal cells, and immune cells [41]. Tumor-associated macrophages (TAM) account for 50% of the TME cell population in some malignancies, such as breast [42], gastric [43], and hepatocellular carcinomas [44]. M1 macrophages, as opposed to M2 macrophages, act as the primary innate host defense force and kill tumor cells [45, 46], thereby inhibiting tumor progression. Tumor cells recruit large numbers of monocytes into the blood and induce them to polarize towards M1 macrophages by secreting relevant cytokines. We found that XCL2, an essential chemokine, plays an important role in M1 macrophage polarization [47, 48]. This shows that XCL2 could be used as a tumor suppressor to inhibit the growth of tumor cells by recruiting M1 macrophages. We also found that cells with high XCL2 expression recruited more M1 macrophages using tissue immunofluorescence staining; *in vitro* experiments revealed that cancer cells were able to recruit more M1 macrophages after XCL2 overexpression in 786O, U251, and MDA-MB-231 cells, which was consistent with the results of our previous bioinformatics analysis. However, more experiments are needed because the specific mechanism of XCL2’s effect on immune cells in the TME is largely unknown.

Given their roles in T cell suppression, anti-PD-1 and anti-PD-L1 have emerged as important hotspots for antibody-based cancer therapy in recent years [49]. To explore the role of immune infiltration in carcinogenesis, we further investigated the expression of XCL2 in all patients who received anti-PD-1 and anti-PD-L1 therapy. The results showed that patients with higher XCL2 expression levels responded better to PD-1 and PD-L1 treatment. This was consistent with our previous findings that XCL2 is a key chemokine in recruiting many immune cells. In the present study, we found that XCL2 could be used as a target for tumor immunotherapy to enhance its efficacy. However, due to a lack of clinical studies with many samples, the molecular mechanism underlying the role of checkpoint XCL2 in cancer development and targeted therapy still needs to be thoroughly investigated.

Recent clinical advances in immune checkpoint inhibitor medicines have pushed immunotherapy out of the highly specialized therapeutic arena and into mainstream oncology [50]. The evolution of our understanding of the mechanisms underlying the immune checkpoint pathway has facilitated the search for pre-treatment and treatment biomarkers [51, 52]. Our study revealed a close relationship between XCL2 and immune checkpoint genes in various human cancers, confirming the hypothesis that XCL2 may enhance immunotherapeutic responses in cancer by synergizing with other known immune checkpoint inhibitors. We also found effects of XCL2 on TMB, MSI, and MMR in the TME. These findings support a possible close link between XCL2, TME, and antitumor immunity.

ScRNA-seq analysis is a powerful tool for characterizing the distribution and functional enrichment of various cell subtypes in the tumor microenvironment [53, 54]. Michael et al. [55] revealed hundreds of molecularly diverse cell types in the nervous system using single-cell sequencing. Furthermore, we used scRNA-seq to characterize the landscape of XCL2 in different cancer cell subsets and found that XCL2 was highly enriched in immune cells, especially CD8+ T cells, NK cells, and macrophages. Meanwhile, the results showed that INF-α and INF-γ responses were primarily enriched in macrophage and CD8+ T cell subtypes in pan-cancer, demonstrating the important role of macrophages and CD8+ T cells in the fight against tumor cells. This was consistent with our previous transcriptome sequencing results.

PPI network mapping in this study provided insight into the role of XCL2, focusing on chemotactic immune cells, in inducing inflammation and immune responses, as well as the complex network of chemokines that interact with each other to jointly participate in the biological functions of the organism. However, we could not identify the different biological functions in the PPI network. However, our subsequent analysis revealed that XCL2 expression could affect apoptosis, cell cycle, DNA damage response, EMT, hormone androgen receptor [56] (hormone AR), hormone ER, PI3K/AKT [57], RTK [58], and TSC/mTOR. Thus, we conclude that XCL2 functions as a chemokine to recruit multiple immune cells and is involved in activating or inhibiting multiple pathways. This greatly complements previous studies [10, 14, 16].

However, our study had some limitations. Firstly, additional cancer types and sample sizes should be added to those listed above. Second, previous studies have shown that XCL1 and XCL2 have similar abilities to recruit various immune cells. In this study, we also demonstrated the chemotactic effect of XCL2 on M1 macrophages using *in vitro* experiments. However, no further *in vitro* studies have been conducted to validate the chemotactic effects of other immune cell types. Therefore, further studies are needed to confirm the complex role of XCL2 in pan-cancers. At the same time, this study found that in some tumors (e.g. KIRC), there was more infiltration of M1-type macrophages with increased XCL2. The survival time of patients was instead reduced, which contradicts the conclusion of our study that patients with high expression of XCL2 should have longer survival due to the anti-tumour effect of M1-type macrophages. This we have not further rationalised in this paper, and we speculate that this is related to macrophage dysfunction, interactions between immune cells, and other factors.

In this study, we found that alterations in XCL2 in cancer cells affect the prognosis of cancer patients, and that said effects may be caused by epigenetic modifications such as DNA methylation and phosphorylation that occur in cancer cells. Considering the function of the XCL2 gene and its important role for the tumour microenvironment in current cancer therapy, we focused our studies on immune cell infiltration. Genes of the chemokine family play a crucial role in this process, and XCL2 is one of them. Chemokines recruit more immune cells, fibroblasts, etc. into the tumour microenvironment (TME) and influence the killing of tumours by strengthening or weakening the local killing effect of the immune system. In this study, we propose that XCL2 is an important chemokine whose high expression affects cancer cells by recruiting M1-type macrophages, followed by an effect that improves the prognosis of cancer patients. This is also a major theoretical basis for our later study on the feasibility of drug therapy. This study provides new ideas for the future field of cancer therapy.

In the future, we will investigate the specific effects of XCL2 on macrophages at a deeper level by interfering with macrophage numbers and function, thus complementing the immunotherapeutic approach in clinical treatment.

## Supplementary Material

Supplementary Figures
