# Supplementary material for: Comprehensive analysis reveals XCL2 as a cancer prognosis and immune infiltration-related biomarker
Source: Aging (Albany NY). 2023 Oct 30;15(21):11891–917. doi: 10.18632/aging.205156 (PMC10683633; doi:10.18632/aging.205156)
Supplement: Supplementary Figures [file aging-15-205156-s001.pdf]

SUPPLEMENTARY FIGURES

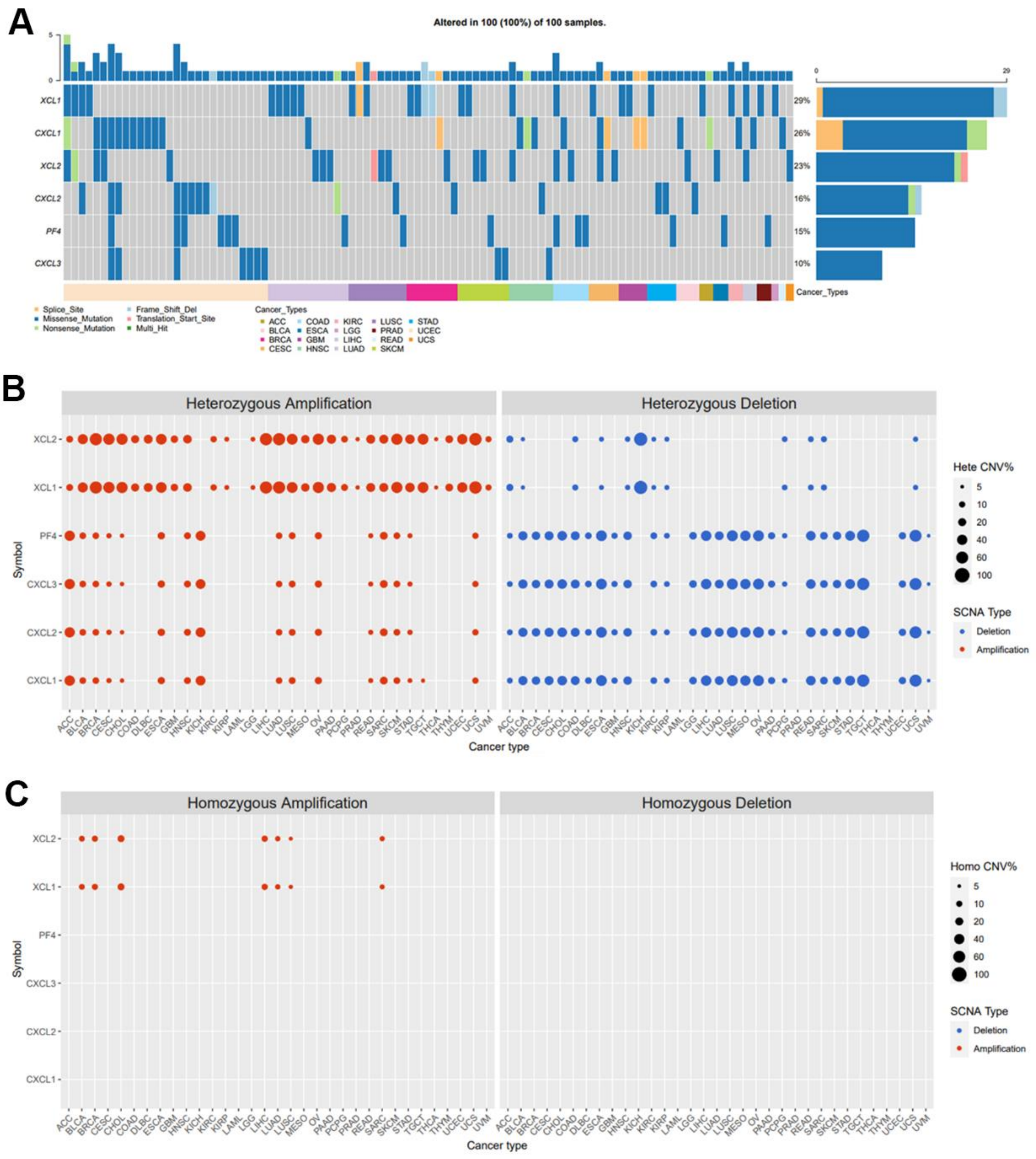

Supplementary Figure 1. The gene mutation character of XCL2 in pan-cancer. (**A**) The SNV rate of XCL2 was 23% in 100 samples and they consisted of splice site, missense mutation and nonsense mutation; (**B**, **C**) The heterozygous amplifications and deletions of XCL2 were common in 33 cancer types; (**D**, **E**) The homozygous amplifications and deletions of XCL2 were common in 33 cancer types.

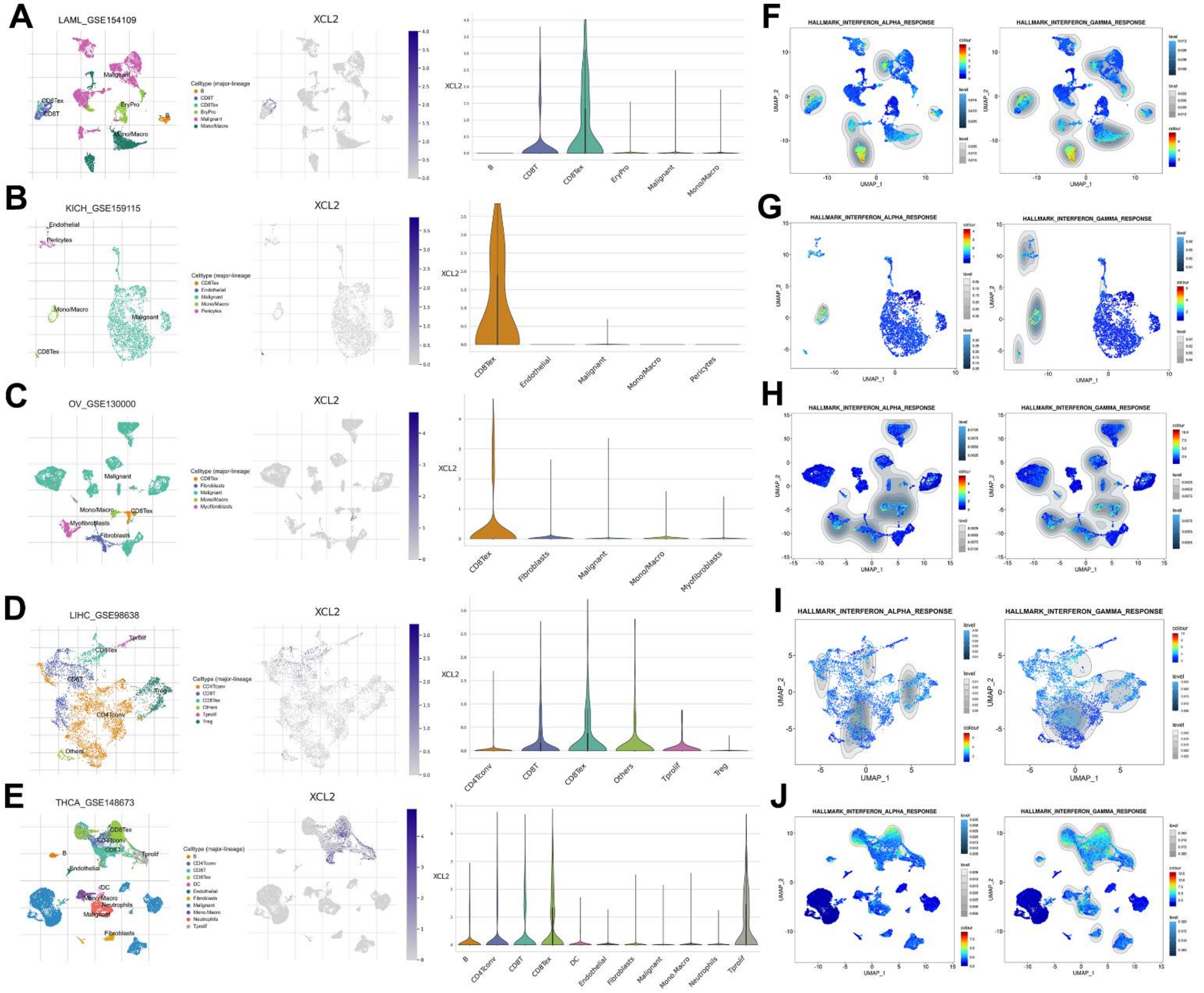

Supplementary Figure 2. The scRNA-seq results of XCL2 expression and single-cell signature explore in pan-cancers. The definition of cancer cells in LAML (**A**), KICH (**B**), OV (**C**), LIHC (**D**), THCA (**E**). Enrichment analysis of interferon alpha response and interferon gamma response in LAML (**F**), KICH (**G**), OV (**H**), LIHC (**I**) and THCA (**J**) single cell sequencing results.

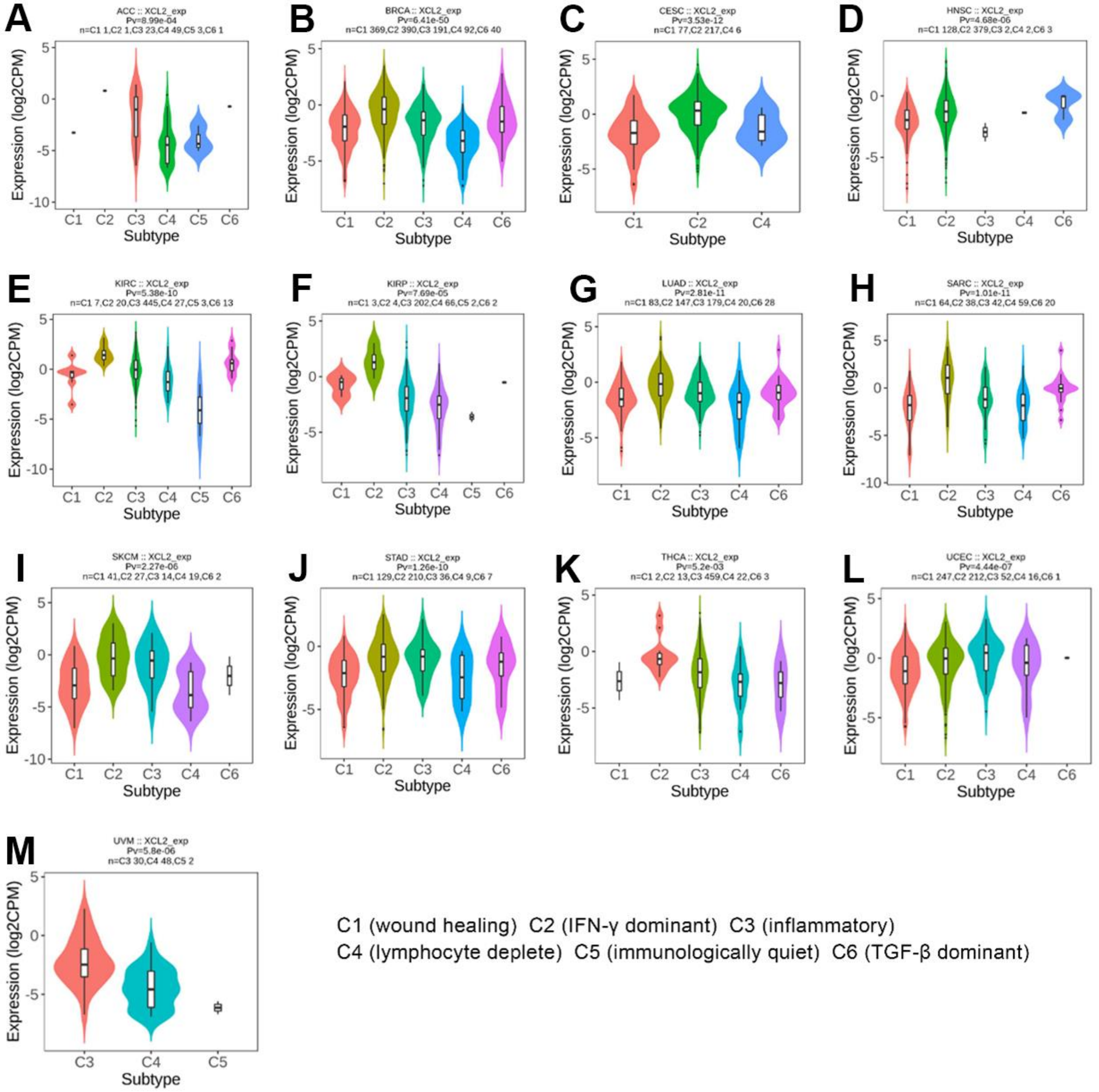

Supplementary Figure 3. Correlations between XCL2 expression and immune subtypes in 13 cancers. (**A**) ACC, (**B**) BRCA, (**C**) CESC, (**D**) HNSC, (**E**) KIRC, (**F**) KIRP, (**G**) LUAD, (**H**) SARC, (**I**) SKCM, (**J**) STAD, (**K**) THCA, (**L**) UCEC, (**M**) UVM. C1 (wound healing), C2 (IFN-g dominant), C3 (inflammatory), C4 (lymphocyte deplete), C5 (immunologically quiet), and C6 (TGF-b dominant).
